# The impact of clinical data on the evaluation of tibial fracture healing

**DOI:** 10.1186/1745-6215-12-237

**Published:** 2011-11-03

**Authors:** Bernadette G Dijkman, Jason W Busse, Stephen D Walter, Mohit Bhandari

**Affiliations:** 1 Division of Orthopaedic Surgery, McMaster University, Hamilton, Ontario, Canada; 2 Department of Clinical Epidemiology and Biostatistics, McMaster University, Hamilton, Ontario, Canada; 3 Institute for Work & Health, Toronto, Ontario, Canada

## Abstract

**Background:**

Radiographic healing is a common outcome measure in orthopedic trials and adjudication by outcome assessors is often conducted on the basis of plain films alone. The degree to which this process reflects clinical practice, in which both plain films and clinical notes are available, is uncertain. We explored the effect of adding clinical notes to radiographs in the adjudication process of a feasibility trial of tibial shaft fractures.

**Methods:**

Radiographic and clinical data from a multicenter randomized controlled trial of 51 patients with operatively treated tibial fractures formed the basis of the study data. At the completion of the trial, serial radiographs (anteroposterior and lateral) were independently evaluated for progression of fracture healing, defined as bridging of at least 3 of 4 cortices, by an adjudication committee comprised of 3 blinded orthopaedic trauma surgeons. Immediately after determination of radiographic time to healing, each surgeon was provided with clinical notes associated with each radiographic follow up visit and asked to re-visit their initial impression. Consensus was achieved for both adjudications. We calculated the percentage of time to healing consensus decisions that changed after evaluation of clinical notes. We further examined the contents of clinical notes and their relative influence on the committee's decisions.

**Results:**

47 of 51 patients were determined to have healed radiographically during the trial follow-up period, and consideration of clinical notes resulted in a change of 40% (19 of 47) of time to healing consensus decisions; however, revised decisions were equally likely to support an earlier or a later time to healing. Clinical notes that resulted in a change to either a 'healed' or a 'not healed' decision contained significantly more comments of either pain resolution or deterioration, respectively, resumption of or failure to resume weightbearing, or either return or no return to work/pre-injury activities (p < 0.001).

**Conclusions:**

The addition of clinical notes to the adjudication of radiographic fracture healing changed the outcome decision in a substantial number of cases. Orthopedic trialists should consider the addition of clinical notes to adjudication material in studies of fracture healing in order to enhance the generalizability of their results.

**Trial Registration:**

The TRUST trial was registered [ID NCT00667849] at http://clinicaltrials.gov/ct2/show/NCT00667849

## Background

Centralized adjudication processes are often used in clinical trials to reduce variation in outcome assessment in which clinical judgment is required [1]. In the case of fracture healing, independent surgeons or radiologists blinded to treatment allocation determine whether a fracture has healed or not [2]. However, orthopedic trials are inconsistent in the information provided to clinicians for adjudication; some provide only plain films or other imaging studies, while others also include clinical notes [3-6].

Fracture healing is a continuous process that is often dichotomized and the decision whether a fracture has healed requires clinical judgment. There is not a standardized approach to the assessment of radiographic fracture healing, and this may influence the results of orthopedic clinical trials [7]. For trial results to be generalizable, the assessment of fracture healing should be consistent with clinical practice. The current study explores the effect of including clinical notes, versus the use of plain films alone, in the adjudication process of fracture healing.

## Methods

The current study was a substudy of a multicenter randomized feasibility trial performed in six level-1 trauma centers across Canada, the eponym for which is TRUST (Trial to Re-evaluate Ultrasound in the Treatment of Tibial Fractures). Each institution's ethics review board approved the study, and we acquired written consent from all patients involved in the study to participate in the trial and to publish their data in aggregate form.

The trial enrolled open (Gustilo Type I-IIIb) or closed (Tscherne Type 0-3) tibial shaft fractures in skeletally mature men and women that were amenable to operative treatment with an intramedullary nail. Patients were followed at 6 weeks, 3, 4, 5, 6, 9 and 12 months after surgery. At each follow up visit, standardized anteroposterior and lateral radiographs were taken. Additionally, surgeons conducted clinical examinations and clinical notes from the visit were obtained. Surgeons were not restricted in the content or length of their clinical notes at each follow up. All radiographs and clinical notes were digitalized and organized per patient and follow up visit on a password-secured website for review by an independent, blinded adjudication committee.

### Adjudication process

Fracture healing was adjudicated centrally and independently by 3 blinded orthopedic trauma surgeons, who were members of a Central Adjudication Committee (CAC). The CAC members gave their opinion whether the fracture was healed or not based on a general assessment of serial radiographs alone. The definition of radiographic healing was defined as full bridging of at least 3 out of 4 cortices. After reaching consensus on the follow up radiograph in which the fracture was healed, each adjudicator reviewed clinical notes associated with each follow up visit. Based on the combination of clinical notes and radiographic information each adjudicator maintained or revised the date of fracture healing, and consensus was achieved through discussion.

### Time to healing decisions

We determined the number of cases in which time to healing was changed after evaluation of clinical notes and the qualitative direction of the change. In addition, we explored the proportion of changed time to healing decisions at each follow up point (6 weeks, 3, 4, 5, 6, 9, and 12 months), since we believe that fracture healing as a continuum has an intermediate stage or "grey zone" when it is particularly challenging for surgeons to make dichotomized decisions based solely on radiographic features [8].

### Evaluation of clinical notes

For decisions about time to fracture healing that changed after examination of clinical information, we examined the contents of the notes. Two reviewers, not involved with the TRUST trial, independently classified content into comments regarding pain, weightbearing, and return to work or pre-injury activities and categorized each comment as positive, negative, or neutral. Positive comments were pain resolution, resumption of weightbearing, and return to work or pre-injury activities, whereas negative comments were characterized by pain deterioration, and failure to resume weightbearing, work, or pre-injury activities. A comment was considered 'neutral' when it was neither decisive of a healed nor of an unhealed fracture, for example "pain is decreasing", "has some pain off and on", or "is walking *fairly *well". In order to compare the proportion of positive, negative, and neutral comments in clinical notes of changed decisions with that of unchanged decisions, we compared all clinical notes associated with changed decisions with a random sample of 100 clinical notes that had not resulted in changed decisions. This sample was obtained by using a computerized random number generator.

### Derivation of Minimally Importance Difference

To understand the potential relevance of our findings, we surveyed a convenience sample of 20 orthopaedic trauma surgeons to determine what proportion of decisions about fracture healing would have to change by the addition of clinical notes to be clinically meaningful. The survey was conducted prior to our analysis. The surgeons were given six pre-determined percentages to choose from: 1%, 5%, 10%, 15%, 20%, and 25%. Among the 17 surgeons who responded, of whom 9 had previously participated in the adjudication processes for a clinical trial of fracture healing, the majority (53%) believed that a ≥ 5% change in decisions about fracture healing when clinical notes were considered constituted an important difference. The remaining surgeons chose thresholds of 10% (2 surgeons), 15% (3), and 20% (2), and one responding surgeon did not choose a percentage as he felt clinical notes were of no use in the adjudication process.

### Statistical Methods

For the calculation of the proportion of time to healing consensus decisions that changed after evaluation of clinical notes, we excluded decisions on cases where radiographic healing had not occurred during the available follow-up data. We used the Pearson χ^2 ^test for comparing proportions of positive, neutral, and negative comments. To evaluate the chance adjusted agreement among the three adjudicators, the kappa (κ) statistic was calculated with confidence intervals of 95%; κ values range from +1, which corresponds to perfect agreement, to -1, corresponding with absolute disagreement. For all analyses, a p-value of < 0.05 was considered as significant. All statistical analyses were performed by SPSS software version 13.0 (*SPSS Inc., Chicago, Illinois*).

## Results

From July 5^th^, 2005, to June 22^nd^, 2007, 51 patients with 14 open and 37 closed tibial fractures were treated with reamed intramedullary nailing. Mean patient age was 39.5 years (standard deviation = 13.6) and 39 patients were male. 47 of 51 patients had follow-up data that allowed for a determination of their time to radiographic healing by the CAC.

### Proportion of changed time to healing decisions

In 28 of 47 patients (60%), the decision on time to healing was conserved, i.e., clinical notes did not result in a change of decision. Of the 19 patients (40%) for whom the addition of clinical notes resulted in a change to the CAC's decision, 9 decisions were changed to support an earlier time to fracture healing, whereas 10 were changed to support a later time to healing. Of the 19 consensus time to healing decisions that were changed after the addition of clinical notes, most occurred at the 3 month (8 decisions; 42%), 4 month (3; 16%), and 5 month (4; 21%) follow up visits (Table 1).

**Table 1:** Proportion of changed time to healing decisions at each follow up point

Follow up	No. of time to healing decisions changed (% of all time to healing decisions)	% of changed time to healing decisions (n = 19)
6 weeks	1 (2.1)	5.3

3 months	8 (17.0)	42.1

4 months	3 (6.4)	15.8

5 months	4 (8.5)	21.1

6 months	1 (2.1)	5.3

9 months	1 (2.1)	5.3

12 months	1 (2.1)	5.3

**Total**	**19 (100)**	**100**

### Interobserver agreement

The agreement among adjudicators was non-significantly higher when the decisions were based on radiographs alone (κ = 0.79, 95% CI 0.70 to 0.88) than when the decisions were based on both radiographs and clinical notes (κ = 0.68, 95% CI 0.59 to 0.77). According to guidelines of Landis and Koch,[9] both these κ values represent substantial interobserver agreement. The interobserver agreement was moderate for the follow up period from 3 to 5 months when decisions were either based on radiographs alone (0.66, 95% CI 0.53 - 0.80), or on a combination of radiographs and clinical information (0.52, 95% CI 0.38 - 0.66). In contrast, agreement was excellent (0.90, 95% CI 0.78 - 1.0 and 0.81, 95% CI 0.68 - 0.93) for the rest of the follow up, respectively, when only radiographs and both radiographs and clinical notes were considered in adjudicators' decisions.

### Comments in clinical notes

**Figure 1:**
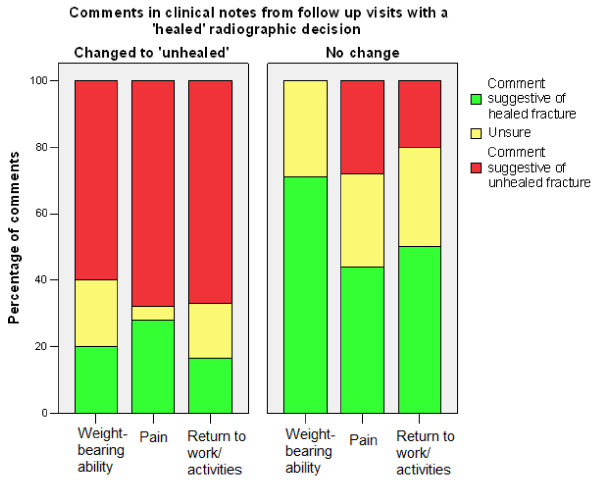
**Comparison of the percentages of comments in clinical notes from follow up visits with a 'healed' radiographic decision that did or did not change the decision to 'unhealed'**.

**Figure 2:**
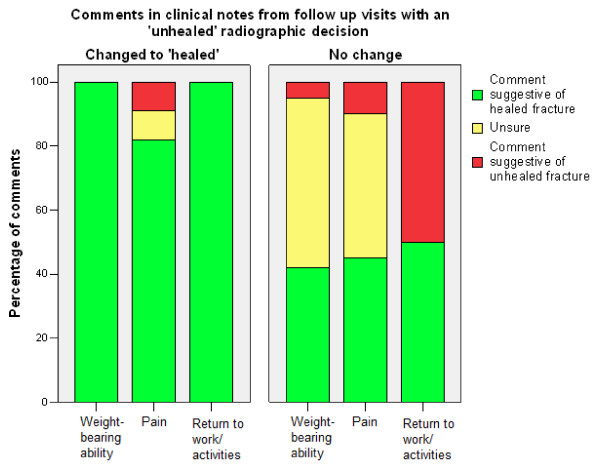
**Comparison of the percentage of comments in clinical notes from follow up visits with an 'unhealed' radiographic decision that did or did not change the decision to 'healed'**.

Clinical notes that changed decisions to 'not healed' mostly contained comments of significant pain and failure to resume weightbearing, work, or pre-injury activities (Table 2, Figure 1). Clinical notes that changed decisions to 'healed' mostly contained comments stating the patient's pain was resolving, and/or that they had resumed weightbearing, or had returned to work or pre-injury activities (Table 2, Figure 2). Clinical notes of follow up visits with unchanged decisions contained fewer positive or negative comments than clinical notes that changed decisions (p < 0.001).

**Table 2:** Comparison of clinical notes' comments between changed and unchanged decisions

		Percentage of comments in radiographically *healed *decisions		Percentage of comments in radiographically *unhealed *decisions	
Content of clinical notes' comments*		**Changed to 'unhealed' after clinical notes**	**No change**	**Changed into 'healed' after clinical notes**	**No change**

**Weightbearing**	Positive	20	71	**100**	**42**
	
	Neutral	20	29	0	53
	
	Negative	**60**	**0**	0	5

**Pain**	Positive	28	44	**82**	**45**
	
	Neutral	4	28	9	45
	
	Negative	**68**	**28**	9	10

**Return to work/leisure activities**	Positive	16.5	50	**100**	**50**
	
	Neutral	16.5	30	0	0
	
	Negative	**67**	**20**	0	50

**Overall**	Positive	21.5	55	**94**	**45.6**
	
	Neural	13.5	29	3	32.6
	
	Negative	**65**	**16**	3	21.6

* A positive content refers to a comment about weightbearing, pain, or return to work/leisure activities which is suggestive of a healed fracture, whereas a negative content suggests that the fracture was not healed yet. A comment was judged to be neutral when it was found neither suggestive of a healed, nor of an unhealed fracture.

## Discussion

In our study, time to healing based on plain films alone was changed in 40% of decisions after the addition of clinical notes. The direction of change was equally distributed between earlier and later healing times, and was associated with the content of clinical notes. Adjudication of fracture healing showed a non-significant trend towards lower agreement when clinical information was considered in addition to plain films. The majority of revisions to time to healing, following the addition of clinical notes, occurred during the 3-5 month follow up period.

Our study does have some limitations. Radiographs for each patient were read twice and the optimal assessment of agreement would have been a paired kappa statistic; however, we were unable to locate a method by which to calculate an associated measure of precision when deriving a paired kappa value for discrete data. Our unpaired analysis would, at most, reduce the power of our comparison, and so our analysis is conservative. Our study dataset was limited to 51 patients, and 3 patients could not be assigned a time to healing based on the available follow up data. Despite our modest patient sample, our study has a number of methodological strengths. First, all outcome assessors in our study had previous experience with outcome adjudication in fracture healing trials. Also, the investigators who judged the content of clinical notes were independent and blinded from the radiographs. Furthermore, the same adjudicators decided on both the radiographic as well as the combined radiographic and clinical fracture healing time, which provides reassurance that the shift in time to healing was solely due to the additional information from clinical notes.

Central adjudication is especially valuable in trials of which outcomes are subjective in their determination [10]. Orthopaedic trials are likely to benefit from central adjudication as fracture healing is a commonly measured outcome which lacks a gold standard and standardized assessment [11]. The use of central adjudication minimizes bias by systematically applying the definition of a study event used in a trial and by assuring that the outcomes assessors are blinded to treatment allocation.

In the current literature, both radiographic and clinical criteria are used to define fracture healing, but no consensus exists about the need for supplementing radiographs with clinical information [7,12,13]. A recent systematic review of 123 trials that evaluated long-bone fracture healing found that the majority (62%) used a combination of radiographic and clinical criteria, but that a substantial proportion (37%) assessed radiographic criteria alone to define fracture healing [7]. It is uncertain to what extent radiographic measures correlate with outcomes that are important to patients, such as pain, the ability to bear weight and return to work or daily activities [13,14].

Including outcome measures that are important to patients in clinical trials is essential. This is reflected by the increased use of patient-based outcomes in clinical trials and the wide variety of available instruments to measure them [15]. Our findings suggest that fracture healing assessment in clinical trials is affected when radiographs are supplemented with clinical notes, which is representative of clinical practice. The effect of supplementing radiographs with clinical information on interobserver agreement is uncertain. Tudor and Taub found that interobserver agreement on various radiographic assessments increased with knowledge of clinical details, [16] whereas Skolasky et al. [17] reported higher disagreement between surgeons (with knowledge of clinical information) and an independent review panel (blinded to clinical information) on spine fusion when imaging studies were supplemented with clinical information. In our study, where the same adjudicators assessed fracture healing based on radiographs alone as with added clinical information, a non-significant decrease in interobserver agreement was seen after the addition of clinical information.

Our findings suggest that the addition of clinical information to radiographs affects the adjudication of fracture healing. Comments in clinical notes regarding weightbearing ability, pain, and return to work or pre-injury activities, which an adjudicator could not possibly obtain from radiographs alone, appear to have influenced adjudicator's decisions. That is, follow up notes that changed a fracture healing decision contained significantly more resolute comments on these outcomes than notes that did not change decisions. Provision of clinical information may bias adjudicators to over- or underinterpret imaging studies; [18] however, we believe that most surgeons in practice use both imaging results and clinical findings to determine healing, and therefore trials that adhere to this practice may provide more generalizeable results.

In order to increase comparability of multiple studies' results, the adjudication process of fracture healing used should be consistent among trials. As the comments regularly included in a clinical note may vary widely between surgeons, a standardized approach of the content of clinical notes, to reflect best practices, may be helpful. We recommend including comments on weightbearing ability and pain in order to maximally approximate the information used to adjudicate fracture healing available in clinical practice.

## Conclusions

The addition of clinical notes to the adjudication of radiographic fracture healing changed the time to healing decision in a substantial number of cases. In order to enhance the generalizability and applicability of clinical studies, orthopaedic trialists should consider adding clinical notes to the adjudication material of fracture healing trials.

## List of Abbreviations

TRUST: Trial to Re-evaluate Ultrasound in the Treatment of Tibial Fractures; CAC: Central Adjudication Committee.

## Declaration of Competing interests

The authors declare that they have no competing interests.

## Authors' contributions

BDG, MB and JWB were responsible for conception and implementation of the study design and data collection. BDG was responsible for design and implementation of the data analysis and prepartion of the manuscript. BDG, MB, JWB and SW were responsible for interpretation of the data. All authors provided critical review of the manuscript.
